# Comment on: The impact of virtual-reality simulation training on operative performance in laparoscopic cholecystectomy: meta-analysis of randomized clinical trials

**DOI:** 10.1093/bjsopen/zrac134

**Published:** 2022-11-22

**Authors:** Conor Toale, Marie Morris, Dara O Kavanagh

**Affiliations:** Department of Surgical Affairs, Royal College of Surgeons in Ireland, Dublin, Ireland; Department of Surgical Affairs, Royal College of Surgeons in Ireland, Dublin, Ireland; Department of Surgical Affairs, Royal College of Surgeons in Ireland, Dublin, Ireland

*Dear Editor*

We read with interest the recent article by Humm *et al*., ‘The impact of virtual-reality simulation training on operative performance in laparoscopic cholecystectomy: meta-analysis of randomized clinical trials’^1^. The authors are to be commended for comprehensively synthesizing the literature demonstrating the impact of simulation training on performance in laparoscopic cholecystectomy.

It is interesting to note that of the five studies that did not record a definitive significant difference in performance scores in favour of virtual-reality simulation training, three did not use a training-to-proficiency model^2–4^, and instead employed time-based curricula. It is possible therefore that participants had received insufficient training to significantly improve performance as measured through objective structured assessment of technical skill or global operative assessment of laparoscopic skills tools. Two of these studies employed medical students with, presumably, very little laparoscopic experience^2,3^. We wonder whether further sub-analysis of studies using a train-to-proficiency model would demonstrate an even greater positive relationship between simulation training and future performance? Furthermore, should participants in all future simulation-training trials train to a predefined, expert standard?

The findings of this review are in keeping with other studies demonstrating the positive impact of simulation training on future performance^5^. Given the potential downstream effects on in-theatre training efficacy, theatre efficiency, and potentially subsequent patient outcomes, the question now is this; what are the institutional, resource, and access factors preventing the widespread adaptation of simulation training curricula in postgraduate surgical training programmes?
